# Supplementary material for: Causal inference concepts can guide research into the effects of climate on infectious diseases
Source: Nat Ecol Evol. 2024 Nov 25;9(2):349–63. doi: 10.1038/s41559-024-02594-3 (PMC11807838; doi:10.1038/s41559-024-02594-3)
Supplement: Supplementary file 1 — Supplementary Methods (Review), Supplementary Tables 1–3 and Supplementary Figs. 1–5. [file 41559_2024_2594_MOESM1_ESM.pdf]

nature ecology & evolution

Supplementary information https://doi.org/10.1038/s41559-024-02594-3

Causal inference concepts can guide research into the effects of climate on infectious diseases

In the format provided by the authors and unedited

Table of Contents

Review of time-series regression studies in environmental epidemiology 2

Supplementary Tables 3

Supplementary Table 1. Review of time-series regression studies in environmental epidemiology 3

Supplementary Table 2: List of weather stations considered for extracting the climatic data 7

Supplementary Table 3: List of model parameters 8

Supplementary figures 9

Supplementary Figure 1. Performance of regression modeling for estimating the causal effects of temperature and relative humidity on the transmission rate (vignette 1) 9

Supplementary Figure 2. Causal graph for the illustrative effect of climate on the effective reproduction number (Re) 10

Supplementary Figure 3. Causal graph for the illustrative confounded effect of climate on spatial spread (vignette 3) 11

Supplementary Figure 4. Confounding bias and how climate variability can masquerade as spatial diffusion in Spain (vignette 3) 12

Supplementary Figure 5. Mediation and the direct and indirect causal effects of temperature on the observed incidence rate (vignette 4) 13

References 14

Review of time-series regression studies in environmental epidemiology

To assess the use of causal inference tools in the field of environmental epidemiology, we reviewed 33 studies—identified in a previous review (1)—that used time-series regression to assess the association between weather and malaria, dengue, cholera, or influenza (Supplementary Table 1). The search strategy and inclusion criteria are outlined in the previous review; of the 33 studies, 9 focused on malaria (2–10), 13 on dengue (11–23), 9 on cholera (24–32), and 2 on influenza (33,34). We then classified every study's objective as causal or predictive based on the occurrence of specific terms to describe its aims or results. The following terms were assumed to represent a:

- causal objective: "effect", "impact", "link", "relationship", "influence", "explain", or "confounding".
- predictive objective: "prediction", "forecasting", or "early warning system".

For every study, we also assessed whether an explicit causal model—for example, in the form of a causal graph—related the weather variables to the outcome time series.

Supplementary Tables

Supplementary Table 1. Review of time-series regression studies in environmental epidemiology
The causal or predictive objective was assessed based on the words underlined (the underlining is ours) in the text quoted from each study. The column 'Causal model?' refers to the presence of an explicit causal model (for example, in the form of a causal graph) relating the weather variables to the outcome time series considered in each study.

Study	Location, period	Pathogen	Objective	Causal model?	Quote
Kim et al., 2012 (6)	Republic of Korea, 2001–2009	Malaria	**Causal**	No	"We aimed to estimate the effects of climatic variables on the transmission of *P. vivax* in temperate regions."
Jusot & Alto, 2011 (5)	Niger, 2000–2003	Malaria	**Causal**	No	"The impact of rainfall 40 days before occurrence of suspected malaria episodes was studied using a distributed lag model."
Haque et al., 2010 (3)	Bangladesh, 1989–2008	Malaria	**Causal**	No	"After adjusting for potential mutual confounding between climatic factors there was no evidence for any association between the number of malaria cases and temperature, rainfall and humidity."
Xiao et al., 2010 (10)	Hainan, China, 1995–2008	Malaria	**Causal**	No	"Cross correlation and autocorrelation analyses were performed to detect the lagged effect of climate factors on malaria transmission and the autocorrelation of malaria incidence."
Olson et al., 2009 (7)	Brazilian Amazon region, 1996–1999	Malaria	**Causal**	No	"Using monthly reports of malaria and precipitation from across the Brazilian Amazon Basin, we demonstrate that malaria incidence and precipitation patterns vary throughout this large region and are influenced by the extent of wetlands."
Hashizume et al., 2009 (4)	Western Kenyan highlands, 1982–2011	Malaria	**Causal**	No	"The purpose of this study was to explore the relationship between the IOD and the incidence of malaria in the western Kenyan highlands, using time-series methodology controlling for the effects of ENSO."
Teklehaimanot et	Ethiopia,	Malaria	**Causal**	No	"The impact of temperature on the duration of a mosquito's life cycle and the sporogonic

Study	Location, period	Pathogen	Objective	Causal model?	Quote
al., 2004 (8)	1990–2000				phase of the parasite could explain the inconsistent findings.”
Teklehaimanot et al., 2004 (9)	Ethiopia, 1990–2000	Malaria	**Predictive**	No	“Here, an attempt to combine these avenues of previous work is described, using modified versions of previously described models based on weather factors to provide predictions of *Plasmodium falciparum* cases in these 10 districts of Ethiopia, and evaluating thresholds that trigger warnings.”
Abeku et al., 2004 (2)	Ethiopia, 1986–1993	Malaria	**Causal**	Yes	“The aim of this study was to quantify the effects of meteorological factors on malaria incidence in areas with unstable transmission using a statistical model based on theoretical reasoning.”
Hii et al., 2012 (15)	Singapore, 2000–2011	Dengue	**Predictive**	No	“The aim of this study was to develop and validate a forecasting model that could predict dengue cases and provide timely early warning in Singapore.”
Gomes et al., 2012 (13)	Rio de Janeiro, Brazil, 2001–2009	Dengue	**Causal**	No	“This study examined the effect of seasonal factors and the relationship between climatic variables and dengue risk in the city of Rio de Janeiro, Brazil, from 2001 to 2009.”
Lowe et al., 2013 (17)	Southeast Brazil, 2001–2009	Dengue	**Predictive**	No	“We illustrate this in the context of developing an early warning system for dengue fever in Southeast Brazil.”
Hashizume et al., 2012 (14)	Dhaka, Bangladesh, 2005–2009	Dengue	**Causal**	No	“We estimated the effects of river levels and rainfall on the hospital admissions for dengue fever at 11 major hospitals in Dhaka, Bangladesh.”
Earnest et al., 2012 (12)	Singapore, 2001–2008	Dengue	**Causal**	No	“The effect of meteorological variables was more pronounced during periods of dengue outbreaks, and this suggests to us that the relationship between weather and dengue is moderated by periods of known dengue outbreaks.”
Pham et al., 2011 (19)	Dak Lak, Vietnam, 2004–2008	Dengue	**Causal**	No	“This study sought to elucidate the linkage between climate factors, mosquito indices and dengue incidence.”

Study	Location, period	Pathogen	Objective	Causal model?	Quote
Pinto et al., 2011 (20)	Singapore, 2000–2007	Dengue	**Causal**	No	"One of the objectives of the study was to estimate the effects of weather on the occurrence of dengue, the relative risk, and the increase of the probability of occurrence using Poisson Regression Model."
Shang et al, 2010 (21)	Taiwan, 1998–2007	Dengue	**Causal**	No	"This study investigated the effect of both imported dengue and local meteorological factors on the occurrence of indigenous dengue in Taiwan."
Chen et al., 2010 (11)	Taiwan, 1998–2008	Dengue	**Causal**	No	"By incorporating the climate variables of current and lagged-time effects of temperature, RH, and rainfall intensity together with BI levels into the Poisson regression model, the impacts of major meteorological factors and mosquito abundance on the dengue fever variability could be quantified."
Tipayamongolgul et al., 2009 (23)	Thailand, 1996–2005	Dengue	**Causal**	No	"Effects of the El Niño-Southern Oscillation on dengue epidemics in Thailand, 1996-2005"
Lu et al., 2009 (18)	Guangzhou, China, 2001–2006	Dengue	**Causal**	No	"This study explored the impact of weather variability on the transmission of dengue fever in the subtropical city of Guangzhou, China."
Johansson et al., 2009 (16)	Puerto Rico, 1986–2006	Dengue	**Causal**	No	"Local and Global Effects of Climate on Dengue Transmission in Puerto Rico"
Thammapalo et al., 2005 (22)	Thailand, 1978–1997	Dengue	**Causal**	No	"The aim of this study was to determine the independent effects of climatic factors: rainfall, temperature and relative humidity, on the occurrence of dengue hemorrhagic fever (DHF) in Thailand after adjustment for cyclical pattern."
Hashizume et al., 2011 (26)	Bangladesh, 1993–2007	Cholera	**Causal**	No	"We explored the relationship between the IOD and the number of cholera patients in Bangladesh, controlling for the effects of ENSO."
Rajendran et al., 2011 (32)	Kolkata, India, 1996–2008	Cholera	**Causal**	No	"The aim of this study was to investigate the relational impact of climate changes on cholera."
Hashizume et al., 2010 (25)	Dhaka, Bangladesh, 1983–2008	Cholera	**Causal**	No	"In summary, seasonal variation in the number of cholera patients in Bangladesh can be explained in part by temperature and rainfall."

Study	Location, period	Pathogen	Objective	Causal model?	Quote
Paz et al., 2009 (31)	Southeast Africa, 1971–2006	Cholera	**Causal**	No	"Impact of Temperature Variability on Cholera Incidence in Southeastern Africa, 1971–2006"
Constantin de Magny et al., 2008 (29)	Matlab, Bangladesh & Kolkata, India, 1998–2006	Cholera	**Predictive**	No	"To determine which environmental signatures were associated with cholera epidemics in Kolkata and in Matlab, and to demonstrate the capacity to predict cholera, a historical approach was taken in modeling the relationship between the dynamics of the cholera epidemics and related environmental factors."
Martinez-Urtaza et al., 2008 (30)	Peru, 1994–2005	Cholera	**Causal**	No	"For the pre–El Niño period, surface seawater temperature was the dominant factor affecting the disease dynamics in the zone. The rise of temperature with the warmer months was the prevalent factor that affected the appearance of infections associated with nonpandemic *V. parahaemolyticus* populations."
Luque Fernandez et al., 2009 (28)	Lusaka, Zambia, 2003–2006	Cholera	**Causal**	No	"The objectives of this study were to describe the evolution of the three cholera epidemics that took place in Lusaka during the period 2003—2006 and to explain the association between the increase in the number of cholera cases on the one hand and daily maximum temperature and rainfall recorded during the period under consideration on the other."
Hashizume et al., 2008 (24)	Dhaka, Bangladesh, 1996–2002	Cholera	**Causal**	No	"This study aimed to investigate the relationship between short-term variations in climate—particularly rainfall—and incidence of cholera in Dhaka, Bangladesh. To gain some insight into possible causal pathways linking rainfall to the occurrence of cholera, the association between river levels and incidence of cholera was also investigated."
Huq et al., 2005 (27)	Bangladesh, 1997–2000	Cholera	**Causal**	No	"The objective of this study was to elucidate the influence of specific environmental factors on outbreaks of cholera, with the ultimate goal being to develop a model for predicting cholera to allow intervention in and/or prevention of cholera epidemics."
Hu et al., 2012 (33)	Brisbane, Australia, 2009	Influenza	**Causal**	No	"This study aimed to use an ecological design to examine the impact of socio-ecological factors and explore the possibility of developing an EWS on the transmission of H1N1 in Brisbane, Australia."
Jusot et al., 2011 (34)	Niger, 2009–2010	Influenza	**Causal**	No	"This work is therefore aimed at studying the link between some climatic factors, particularly temperature and humidity, and the occurrence of influenza in Niamey."

Supplementary Table 2: List of weather stations considered for extracting the climatic data

Country	Weather station name	Weather station location (longitude, latitude)	Nearest major city
Germany	EDHL	(10.7, 53.8)	Lübeck
Spain	LEAS	(-6.0, 43.5)	Gijón
Spain	LEXJ	(-3.8, 43.4)	Santander
Spain	LEBB	(-2.9, 43.2)	Bilbao
Spain	LEPP	(-1.6, 42.8)	Pamplona
Spain	LELO	(-2.3, 42.5)	Logroño
Spain	LEVX	(-8.6, 42.2)	Vigo
Spain	LEVD	(-4.8, 41.7)	Valladolid
Spain	LEZG	(-1.0, 41.7)	Zaragoza
Spain	LEBL	(2.1, 41.3)	Barcelona
Spain	LEVS	(-3.6, 40.5)	Madrid
Spain	LEPA	(2.7, 39.5)	Palma
Spain	LEVC	(-0.5, 39.5)	Valencia
Spain	LEAB	(-1.9, 39.0)	Albacete
Spain	LEBZ	(-6.8, 38.9)	Badajoz
Spain	LEZL	(-5.9, 37.4)	Seville
Colombia	SKRH	(-72.9, 11.5)	Riohacha
Colombia	SKSM	(-74.2, 11.1)	Santa Marta
Colombia	SKBQ	(-74.8, 10.9)	Barranquilla
Colombia	SKCG	(-75.5, 10.5)	Cartagena
Colombia	SKVP	(-73.2, 10.4)	Valledupar
Colombia	SKMR	(-75.8, 8.8)	Montería
Colombia	SKCC	(-72.5, 7.9)	Cúcuta
Colombia	SKUC	(-70.7, 7.1)	Arauca
Colombia	SKMD	(-75.6, 6.3)	Medellín
Colombia	SKPC	(-67.5, 6.2)	Puerto Carreño
Colombia	SKUI	(-76.6, 5.7)	Quibdó
Colombia	SKPE	(-75.7, 4.8)	Pereira
Colombia	SKBO	(-74.1, 4.7)	Bogotá
Colombia	SKAR	(-75.8, 4.5)	Ibagué
Colombia	SKVV	(-73.6, 4.2)	Villavicencio
Colombia	SKCL	(-76.4, 3.5)	Cali
Colombia	SKNV	(-75.3, 2.9)	Neiva
Colombia	SKPS	(-77.3, 1.4)	Pasto
Colombia	SKLT	(-69.9, -4.2)	Leticia

Supplementary Table 3: List of model parameters

Parameter	Meaning	Value(s)	Comment
μ	Birth/death rate	1/80 per year	Population size assumed constant
N	Population size	5M	—
$R_0 = \frac{\beta}{\mu+1}$	Basic reproduction number	Vignette 1: 1.25, 2.5, 5<br>Vignette 2: 2.5<br>Vignettes 3–4: 1.25	β: Average transmission rate
$1/\alpha$	Average duration of protection	Vignette 1, 3, 4: 1 yr<br>Vignette 2: 2 yr	—
γ^{-1}	Generation time	1 week	Assumed fixed and equal to the time step
δ_{Te}	Effect of temperature on transmission	Vignettes 1–3: –0.2<br>Vignette 4: 0 or –0.2	Small negative effect assumed (35,36)
δ_{RH}	Effect of temperature on relative humidity	–0.2	Small negative effect assumed (35,36)
$\bar{\rho}$	Average reporting probability	0.1	—
ρ_k	Reporting overdispersion (Negative-Binomial model)	Vignettes 1, 3, 4: 0.04	Coefficient of variation of observation model $\approx \sqrt{\rho_k} = 20\%$ (37)

Supplementary figures

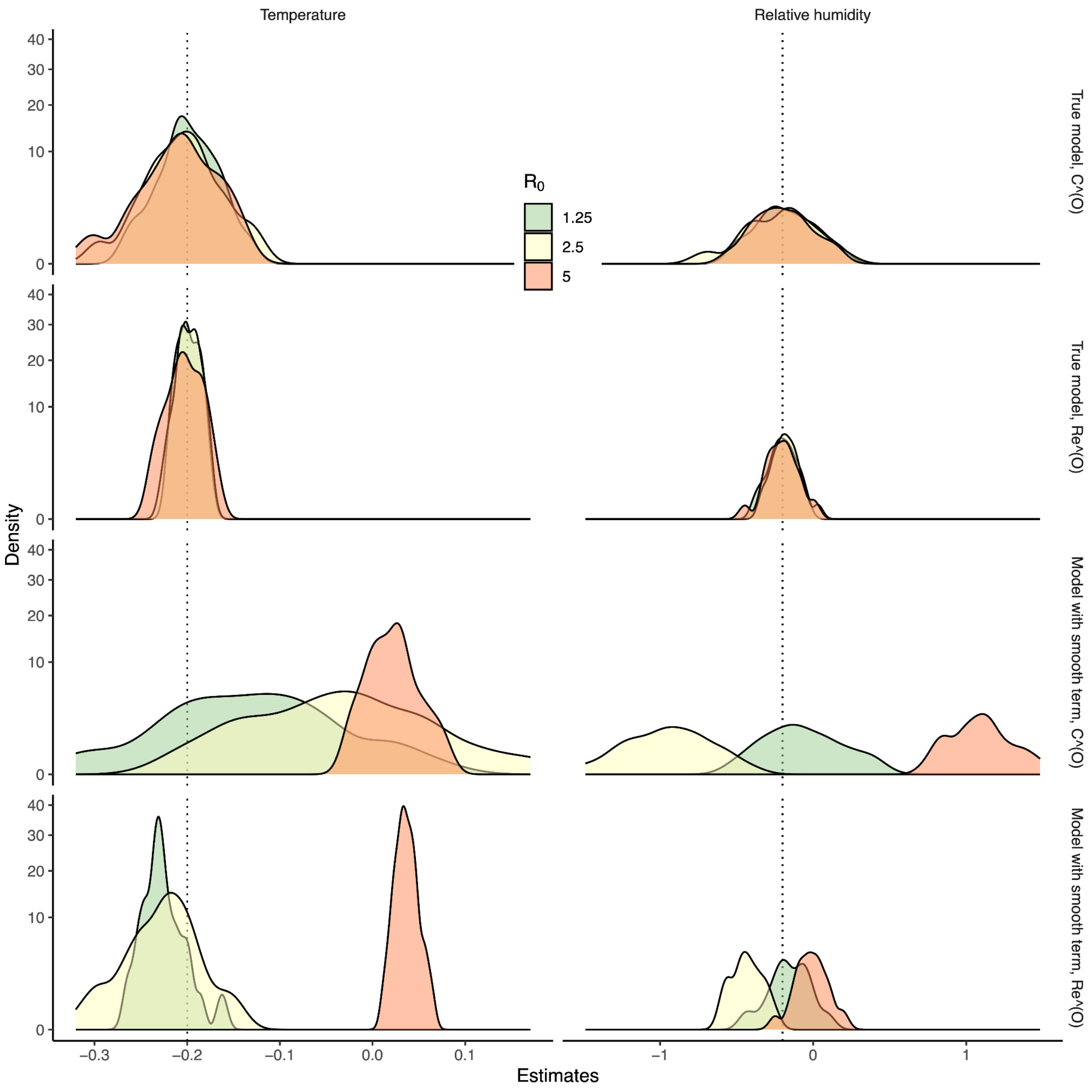

Supplementary Figure 1. Performance of regression modeling for estimating the causal effects of temperature and relative humidity on the transmission rate (vignette 1)

Plotted is the distribution of regression coefficients estimated from Negative Binomial GAMs fitted to 100 replicate time series of the observed incidence rate (denoted by C^(O)) or the observed effective reproduction number (denoted by Re^(O)). The vertical dotted line indicates the true effect fixed in the transmission model for generating the observations ($\delta_{Te} = \delta_{RH} = -0.2$). From top to bottom, the models differ in the covariate included in addition to the environmental variables: $\log(S_{t-1} I_{t-1})$ for the first model, $\log S_t$ for the second, and a smooth of time for the last two.

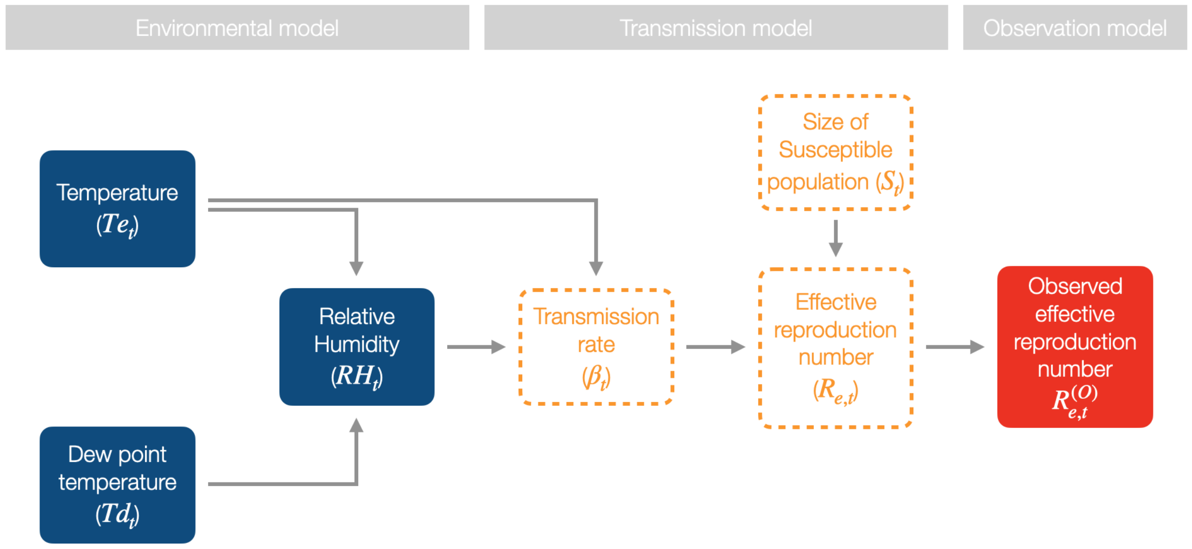

Supplementary Figure 2. Causal graph for the illustrative effect of climate on the effective reproduction number (Re)

Another outcome considered to assess the effect of weather is the effective reproduction number. In contrast to the observed incidence rate, the effective reproduction number depends on just one unobserved variable (S_t). Variables surrounded by dashed lines are assumed to be unobserved.

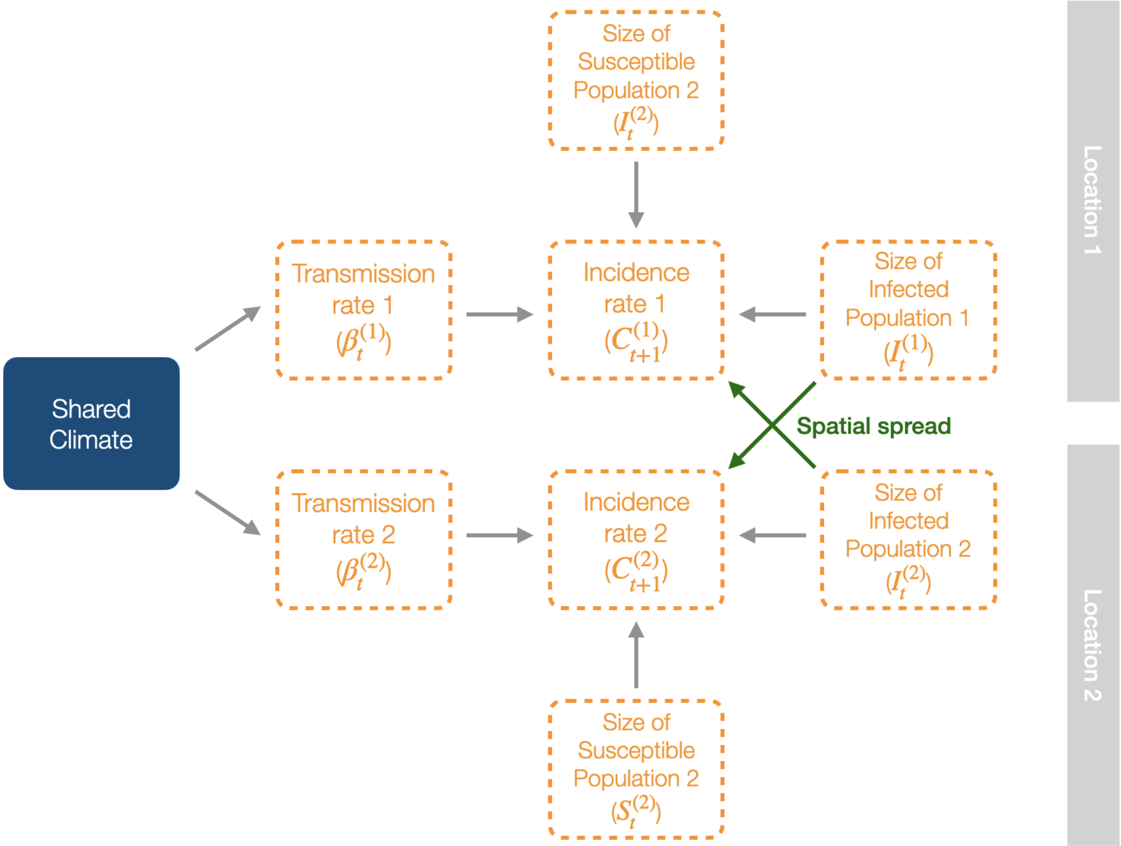

Supplementary Figure 3. Causal graph for the illustrative confounded effect of climate on spatial spread (vignette 3)

Spatial patterns in infectious diseases can arise from the movement of infectees—spatial spread—from one location to another (in green). Climatic variables (*e.g.*, temperature or relative humidity) affecting transmission across locations may confound the effect of spatial spread through the path: Incidence rate at location 1 ($C_{t+1}^{(1)}$) ← Climate → Incidence rate at location 2 ($C_{t+1}^{(2)}$).

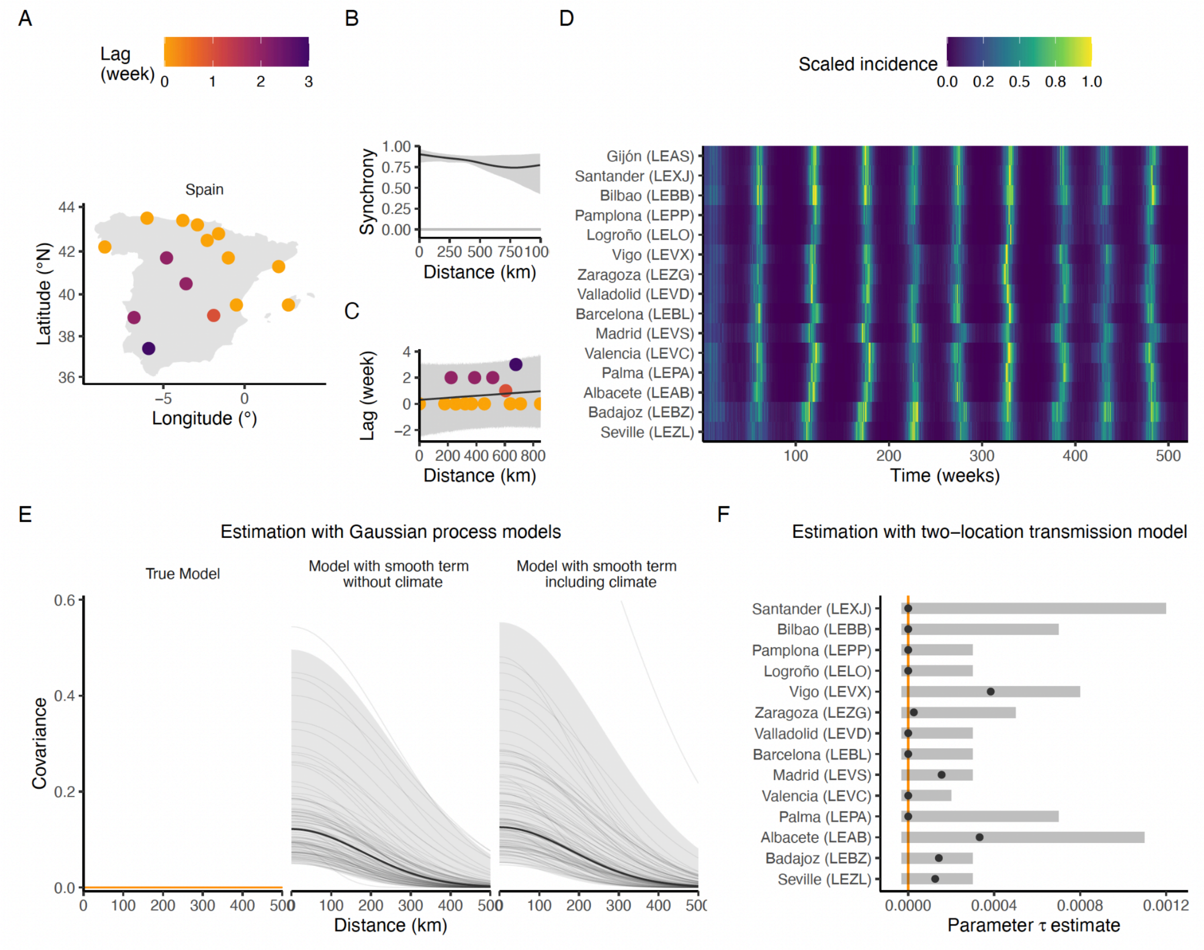

Supplementary Figure 4. Confounding bias and how climate variability can masquerade as spatial diffusion in Spain (vignette 3)

A: Considering 15 locations across Spain, we D: simulated incidence with no spatial diffusion but a common effect of climate on transmission (see Methods for full details). B: Pairwise epidemic synchrony between locations, with the fitted covariance function shown as a line and the 95% confidence interval shaded in grey. C: Relative timing of the simulated epidemic peaks across Spain, referenced to the northernmost location (Gijón). The color indicates the time difference between the epidemic peaks of each site and those of the reference site. The line represents the line posterior mean from a Bayesian linear model, from which we estimated the traveling wave, and the gray envelope represents the 95% credible interval. E: Estimated spatial spread from Gaussian process models, with the dark lines representing the estimated mean covariance function, light lines representing 100 draws from the posterior distribution, and shaded envelopes (orange and grey) indicating the 95% credible interval. F: Estimated spatial spread from two-location transmission models assuming no effect of climate, where the orange line denotes the true value of spatial spread, $\tau = 0$. The points represent the maximum likelihood estimate of the spatial spread, and the gray intervals represent the 95% confidence interval. Model parameters: basic reproduction number of 2.5, average duration of immunity of two years, and other parameters as in Supplementary Table 3.

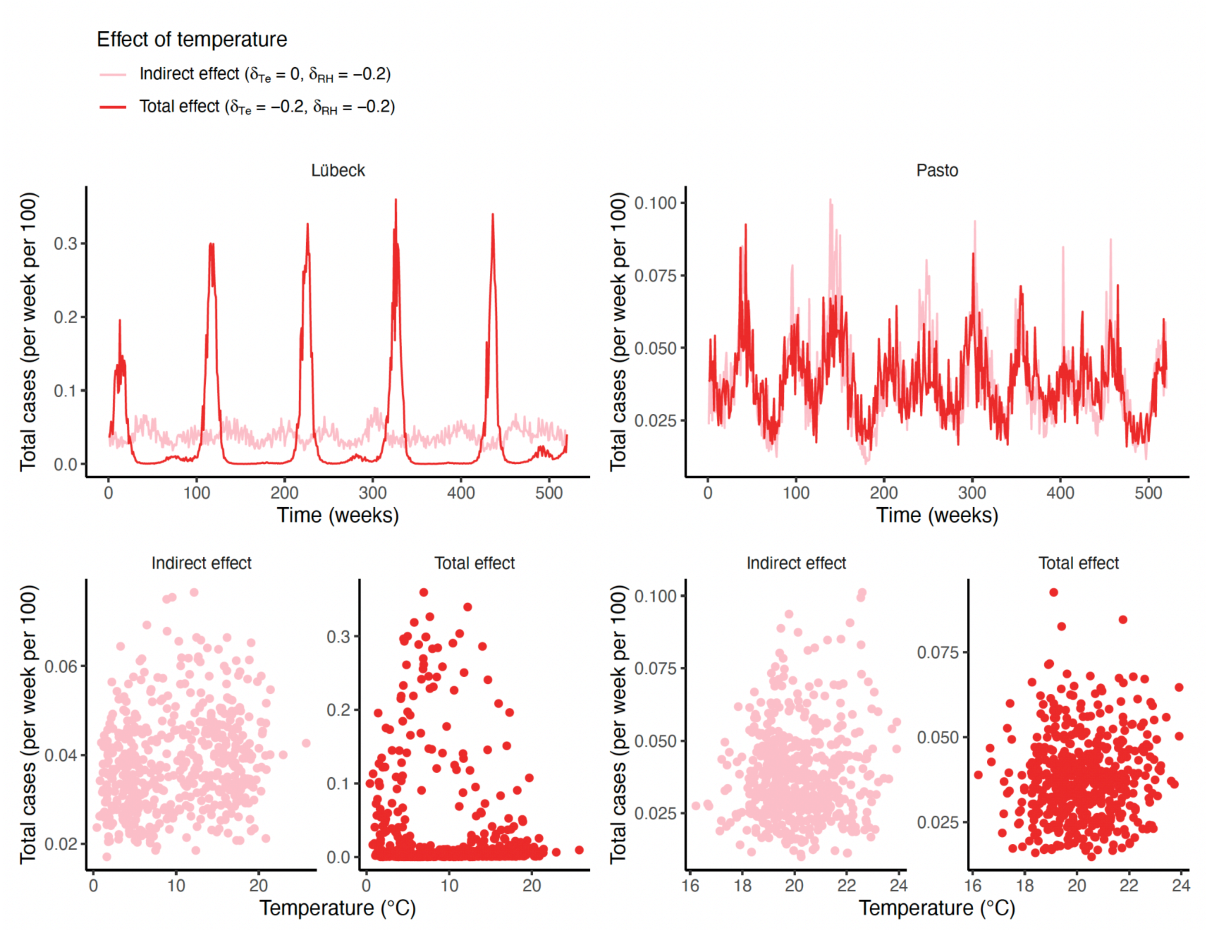

Supplementary Figure 5. Mediation and the direct and indirect causal effects of temperature on the observed incidence rate (vignette 4)

Simulated observed incidence rate (top panels) in A: Lübeck, Germany, and B: Pasto, Colombia, from models including the total effect of temperature (dark lines, where the direct effect of temperature, δ_{TE}, and the direct effect of RH, δ_{RH}, were –0.2) or the indirect effect of temperature mediated through humidity (light lines, where $\delta_{TE} = 0$ and $\delta_{RH} =- 0.2$, see Methods for full details). The bottom panels show the indirect effect of temperature through relative humidity on the observed incidence rate (left panels) and the total effect of temperature (right panels). Model parameters: basic reproduction number of 1.25, average duration of immunity of one year, and other parameters as in Supplementary Table 3.

References

1. Imai C, Hashizume M. A systematic review of methodology: time series regression analysis for environmental factors and infectious diseases. Trop Med Health. 2015 Mar;43(1):1–9.

2. Abeku TA, De Vlas SJ, Borsboom GJJM, Tadege A, Gebreyesus Y, Gebreyohannes H, et al. Effects of meteorological factors on epidemic malaria in Ethiopia: a statistical modelling approach based on theoretical reasoning. Parasitology. 2004 Jun;128(Pt 6):585–93.

3. Haque U, Hashizume M, Glass GE, Dewan AM, Overgaard HJ, Yamamoto T. The role of climate variability in the spread of malaria in Bangladeshi highlands. PLoS One. 2010 Dec 16;5(12):e14341.

4. Hashizume M, Terao T, Minakawa N. The Indian Ocean Dipole and malaria risk in the highlands of western Kenya. Proc Natl Acad Sci U S A. 2009 Feb 10;106(6):1857–62.

5. Jusot JF, Alto O. Short term effect of rainfall on suspected malaria episodes at Magaria, Niger: a time series study. Trans R Soc Trop Med Hyg. 2011 Nov;105(11):637–43.

6. Kim YM, Park JW, Cheong HK. Estimated effect of climatic variables on the transmission of Plasmodium vivax malaria in the Republic of Korea. Environ Health Perspect. 2012 Sep;120(9):1314–9.

7. Olson SH, Gangnon R, Elguero E, Durieux L, Guégan JF, Foley JA, et al. Links between climate, malaria, and wetlands in the Amazon Basin. Emerg Infect Dis. 2009 Apr;15(4):659–62.

8. Teklehaimanot HD, Lipsitch M, Teklehaimanot A, Schwartz J. Weather-based prediction of Plasmodium falciparum malaria in epidemic-prone regions of Ethiopia I. Patterns of lagged weather effects reflect biological mechanisms. Malar J. 2004 Nov 12;3:41.

9. Teklehaimanot HD, Schwartz J, Teklehaimanot A, Lipsitch M. Weather-based prediction of Plasmodium falciparum malaria in epidemic-prone regions of Ethiopia II. Weather-based prediction systems perform comparably to early detection systems in identifying times for interventions. Malar J. 2004 Nov 19;3:44.

10. Xiao D, Long Y, Wang S, Fang L, Xu D, Wang G, et al. Spatiotemporal distribution of malaria and the association between its epidemic and climate factors in Hainan, China. Malar J. 2010 Jun 25;9:185.

11. Chen SC, Liao CM, Chio CP, Chou HH, You SH, Cheng YH. Lagged temperature effect with mosquito transmission potential explains dengue variability in southern Taiwan: insights from a statistical analysis. Sci Total Environ. 2010 Sep 1;408(19):4069–75.

12. Earnest A, Tan SB, Wilder-Smith A. Meteorological factors and El Niño Southern Oscillation are independently associated with dengue infections. Epidemiol Infect. 2012 Jul;140(7):1244–51.

13. Gomes AF, Nobre AA, Cruz OG. Temporal analysis of the relationship between dengue and meteorological variables in the city of Rio de Janeiro, Brazil, 2001-2009. Cad Saude Publica. 2012 Nov;28(11):2189–97.

14. Hashizume M, Dewan AM, Sunahara T, Rahman MZ, Yamamoto T. Hydroclimatological variability and dengue transmission in Dhaka, Bangladesh: a time-series study. BMC Infect Dis. 2012 Apr 24;12:98.

15. Hii YL, Zhu H, Ng N, Ng LC, Rocklöv J. Forecast of dengue incidence using temperature and rainfall. PLoS Negl Trop Dis. 2012 Nov 29;6(11):e1908.

16. Johansson MA, Dominici F, Glass GE. Local and global effects of climate on dengue transmission in Puerto Rico. PLoS Negl Trop Dis. 2009 Feb 17;3(2):e382.

17. Lowe R, Bailey TC, Stephenson DB, Jupp TE, Graham RJ, Barcellos C, et al. The development of an early warning system for climate-sensitive disease risk with a focus on dengue epidemics in Southeast Brazil. Stat Med. 2013 Feb 28;32(5):864–83.

18. Lu L, Lin H, Tian L, Yang W, Sun J, Liu Q. Time series analysis of dengue fever and weather in Guangzhou, China. BMC Public Health. 2009 Oct 27;9:395.

19. Pham HV, Doan HTM, Phan TTT, Minh NNT. Ecological factors associated with dengue fever in a Central Highlands province, Vietnam. BMC Infect Dis. 2011 Jun 16;11:172.

20. Pinto E, Coelho M, Oliver L, Massad E. The influence of climate variables on dengue in Singapore. Int J Environ Health Res. 2011 Dec;21(6):415–26.

21. Shang CS, Fang CT, Liu CM, Wen TH, Tsai KH, King CC. The role of imported cases and favorable meteorological conditions in the onset of dengue epidemics. PLoS Negl Trop Dis. 2010 Aug 3;4(8):e775.

22. Thammapalo S, Chongsuwiwatwong V, McNeil D, Geater A. The climatic factors influencing the occurrence of dengue hemorrhagic fever in Thailand. Southeast Asian J Trop Med Public Health. 2005 Jan;36(1):191–6.

23. Tipayamongkholgul M, Fang CT, Klinchan S, Liu CM, King CC. Effects of the El Niño-southern oscillation on dengue epidemics in Thailand, 1996-2005. BMC Public Health. 2009 Nov 20;9:422.

24. Hashizume M, Armstrong B, Hajat S, Wagatsuma Y, Faruque ASG, Hayashi T, et al. The effect of rainfall on the incidence of cholera in Bangladesh. Epidemiology. 2008 Jan;19(1):103–10.

25. Hashizume M, Faruque ASG, Wagatsuma Y, Hayashi T, Armstrong B. Cholera in Bangladesh: climatic components of seasonal variation. Epidemiology. 2010 Sep;21(5):706–10.

26. Hashizume M, Faruque ASG, Terao T, Yunus M, Streatfield K, Yamamoto T, et al. The Indian Ocean dipole and cholera incidence in Bangladesh: a time-series analysis. Environ Health Perspect. 2011 Feb;119(2):239–44.

27. Huq A, Sack RB, Nizam A, Longini IM, Nair GB, Ali A, et al. Critical factors influencing the occurrence of Vibrio cholerae in the environment of Bangladesh. Appl Environ Microbiol. 2005 Aug;71(8):4645–54.

28. Luque Fernández MA, Bauernfeind A, Jiménez JD, Gil CL, El Omeiri N, Guibert DH. Influence of temperature and rainfall on the evolution of cholera epidemics in Lusaka, Zambia, 2003-2006: analysis of a time series. Trans R Soc Trop Med Hyg. 2009 Feb;103(2):137–43.

29. Constantin de Magny G, Murtugudde R, Sapiano MRP, Nizam A, Brown CW, Busalacchi AJ, et al. Environmental signatures associated with cholera epidemics. Proc Natl Acad Sci U S A. 2008 Nov 18;105(46):17676–81.

30. Martinez-Urtaza J, Huapaya B, Gavilan RG, Blanco-Abad V, Ansede-Bermejo J, Cadarso-Suarez C, et al. Emergence of Asiatic Vibrio diseases in South America in phase with El Niño. Epidemiology. 2008 Nov;19(6):829–37.

31. Paz S. Impact of temperature variability on cholera incidence in southeastern Africa, 1971-2006. Ecohealth. 2009 Sep;6(3):340–5.

32. Rajendran K, Sumi A, Bhattachariya MK, Manna B, Sur D, Kobayashi N, et al. Influence of relative humidity in Vibrio cholerae infection: a time series model. Indian J Med Res. 2011 Feb;133(2):138–45.

33. Hu W, Williams G, Phung H, Birrell F, Tong S, Mengersen K, et al. Did socio-ecological factors drive the spatiotemporal patterns of pandemic influenza A (H1N1)? Environ Int. 2012 Sep 15;45:39–43.

34. Jusot JF, Adamou L, Collard JM. Influenza transmission during a one-year period (2009-2010) in a Sahelian city: low temperature plays a major role. Influenza Other Respi Viruses. 2012 Mar;6(2):87–9.

35. Morris DH, Yinda KC, Gamble A, Rossine FW, Huang Q, Bushmaker T, et al. Mechanistic theory predicts the effects of temperature and humidity on inactivation of SARS-CoV-2 and other enveloped viruses. Elife [Internet]. 2021 Jul 13;10. Available from: http://dx.doi.org/10.7554/eLife.65902

36. Prussin AJ 2nd, Schwake DO, Lin K, Gallagher DL, Buttling L, Marr LC. Survival of the Enveloped Virus Phi6 in Droplets as a Function of Relative Humidity, Absolute Humidity, and Temperature. Appl Environ Microbiol [Internet]. 2018 Jun 15;84(12). Available from: http://dx.doi.org/10.1128/AEM.00551-18

37. Domenech de Cellès M, Wong A, Andrea Barrero Guevara L, Rohani P. Immunological heterogeneity informs estimation of the durability of vaccine protection. J R Soc Interface. 2022 May;19(190):20220070.
