# Supplementary material for: Causal inference concepts can guide research into the effects of climate on infectious diseases
Source: Nat Ecol Evol. 2024 Nov 25;9(2):349–63. doi: 10.1038/s41559-024-02594-3 (PMC11807838; doi:10.1038/s41559-024-02594-3)
Supplement: Supplementary file 2 — Reporting Summary [file 41559_2024_2594_MOESM2_ESM.pdf]

nature portfolio

Corresponding author(s): Dr. Matthieu Domenech de Cellès
Last updated by author(s): Oct 25, 2024

Reporting Summary

Nature Portfolio wishes to improve the reproducibility of the work that we publish. This form provides structure for consistency and transparency in reporting. For further information on Nature Portfolio policies, see our Editorial Policies and the Editorial Policy Checklist.

Statistics

For all statistical analyses, confirm that the following items are present in the figure legend, table legend, main text, or Methods section.

n/a	Confirmed	
☐	☒	The exact sample size (*n*) for each experimental group/condition, given as a discrete number and unit of measurement
☐	☒	A statement on whether measurements were taken from distinct samples or whether the same sample was measured repeatedly
☐	☒	The statistical test(s) used AND whether they are one- or two-sided<br>*Only common tests should be described solely by name; describe more complex techniques in the Methods section.*
☐	☒	A description of all covariates tested
☐	☒	A description of any assumptions or corrections, such as tests of normality and adjustment for multiple comparisons
☐	☒	A full description of the statistical parameters including central tendency (e.g. means) or other basic estimates (e.g. regression coefficient) AND variation (e.g. standard deviation) or associated estimates of uncertainty (e.g. confidence intervals)
☒	☐	For null hypothesis testing, the test statistic (e.g. *F*, *t*, *r*) with confidence intervals, effect sizes, degrees of freedom and *P* value noted<br>*Give P values as exact values whenever suitable.*
☐	☒	For Bayesian analysis, information on the choice of priors and Markov chain Monte Carlo settings
☒	☐	For hierarchical and complex designs, identification of the appropriate level for tests and full reporting of outcomes
☐	☒	Estimates of effect sizes (e.g. Cohen's *d*, Pearson's *r*), indicating how they were calculated

Our web collection on statistics for biologists contains articles on many of the points above.

Software and code

Policy information about availability of computer code

Data collection	The simulated data was generated from a mathematical model of transmission using the package pomp (version 5.11) in R (version 4.4.1). The daily average records of dew point temperature (Td, expressed in °C) and ambient temperature (Te, expressed in °C) were extracted using the WeatherData function in Mathematica (version 12.0).
Data analysis	All analyses were conducted in R (version 4.4.1). For reproducibility, the renv package (version 1.0.7) was used to keep track of all the packages' version. The model was coded and run using the package pomp (version 5.11). Further analyses were performed using the packages mgcv (version 1.9-1), brms (version 2.21.0), and ncf (version 1.3-2). All code can be found at: https://github.com/DomenechLab/Causality_Seasonality and are stored in Edmond, the open research data repository of the Max Planck Society, https://doi.org/10.17617/3.9CWN7W.

For manuscripts utilizing custom algorithms or software that are central to the research but not yet described in published literature, software must be made available to editors and reviewers. We strongly encourage code deposition in a community repository (e.g. GitHub). See the Nature Portfolio guidelines for submitting code & software for further information.

Data

Policy information about availability of data

All manuscripts must include a data availability statement. This statement should provide the following information, where applicable:

- Accession codes, unique identifiers, or web links for publicly available datasets
- A description of any restrictions on data availability
- For clinical datasets or third party data, please ensure that the statement adheres to our policy

All data and R programming codes are available at https://github.com/DomenechLab/Causality_Seasonality and stored in Edmond, the open research data repository of the Max Planck Society, https://doi.org/10.17617/3.9CWN7W.

Research involving human participants, their data, or biological material

Policy information about studies with human participants or human data. See also policy information about sex, gender (identity/presentation), and sexual orientation and race, ethnicity and racism.

Reporting on sex and gender	NA
Reporting on race, ethnicity, or other socially relevant groupings	NA
Population characteristics	NA
Recruitment	NA
Ethics oversight	NA

Note that full information on the approval of the study protocol must also be provided in the manuscript.

Field-specific reporting

Please select the one below that is the best fit for your research. If you are not sure, read the appropriate sections before making your selection.

☐ Life sciences ☐ Behavioural & social sciences ☒ Ecological, evolutionary & environmental sciences

For a reference copy of the document with all sections, see nature.com/documents/nr-reporting-summary-flat.pdf

Ecological, evolutionary & environmental sciences study design

All studies must disclose on these points even when the disclosure is negative.

Study description	This study explores how concepts from causal inference can be applied to understand the effects of weather on the transmission and infection dynamics of infectious diseases in the context of climate change. We use mathematical models of transmission to generate simulations and demonstrate how these concepts improve study design, reduce bias, and enhance the interpretation of the impacts of climatic variables on disease transmission.
Research sample	We generated simulations from a mathematical model of transmission forced by weather (temperature and relative humidity). We used weather data from major cities of Colombia, Spain, and Germany. We chose these locations according to the characteristic of the climate.
Sampling strategy	This is a simulation study that used previously-collected climatic data, we did not conduct any sampling.
Data collection	The climatic data was collected from multiple weather stations located near the major cities of Colombia, Spain, and Germany.
Timing and spatial scale	The simulations were generated using weekly climatic data covering the period 2013–2022 in multiple weather stations located near the major cities of Colombia, Spain, and Germany.
Data exclusions	We did not excluded data for this study.
Reproducibility	All code to simulate the data and repeat the analyses has been made publicly available and the renv package (version 1.0.7) was used to keep track of all the packages' version.
Randomization	We simulated data from a mathematical model of transmission; no randomization was conducted.
Blinding	This is a modeling study, in which we used a transmission model to simulate data; blinding is not relevant to this study design.

Did the study involve field work? ☐ Yes ☒ No

Reporting for specific materials, systems and methods

We require information from authors about some types of materials, experimental systems and methods used in many studies. Here, indicate whether each material, system or method listed is relevant to your study. If you are not sure if a list item applies to your research, read the appropriate section before selecting a response.

Materials & experimental systems

n/a	Involved in the study
☒	☐ Antibodies
☒	☐ Eukaryotic cell lines
☒	☐ Palaeontology and archaeology
☒	☐ Animals and other organisms
☒	☐ Clinical data
☒	☐ Dual use research of concern
☒	☐ Plants

Methods

n/a	Involved in the study
☒	☐ ChIP-seq
☒	☐ Flow cytometry
☒	☐ MRI-based neuroimaging

Plants

Seed stocks	NA
Novel plant genotypes	NA
Authentication	NA
